# A Novel Design of a 3D Racetrack Memory Based on Functional Segments in Cylindrical Nanowire Arrays

**DOI:** 10.3390/nano10122403

**Published:** 2020-12-01

**Authors:** Javier Rial, Mariana P. Proenca

**Affiliations:** 1 IFIMUP—Institute of Physics for Advanced Materials, Nanotechnology and Photonics, Department of Physics and Astronomy, Faculty of Sciences, University of Porto, Rua do Campo Alegre 678, 4169-007 Porto, Portugal; 2 ISOM—Institute of Optoelectronic Systems and Microtechnology, Technical University of Madrid, Avda. Complutense 30, 28040, Madrid, Spain

**Keywords:** domain wall, cylindrical nanowire arrays, micromagnetic simulations, racetrack memories, multi-segmented nanowires, diameter-modulated nanowires

## Abstract

A racetrack memory is a device where the information is stored as magnetic domains (bits) along a nanowire (track). To read and record the information, the bits are moved along the track by current pulses until they reach the reading/writing heads. In particular, 3D racetrack memory devices use arrays of vertically aligned wires (tracks), thus enhancing storage density. In this work, we propose a novel 3D racetrack memory configuration based on functional segments inside cylindrical nanowire arrays. The innovative idea is the integration of the writing element inside the racetrack itself, avoiding the need to implement external writing heads next to the track. The use of selective magnetic segments inside one nanowire allows the creation of writing and storage sections inside the same track, separated by chemical constraints identical to those separating the bits. Using micromagnetic simulations, our study reveals that if the writing section is composed of two segments with different coercivities, one can reverse its magnetization independently from the rest of the memory device by applying an external magnetic field. Spin-polarized current pulses then move the information bits along selected tracks, completing the writing process by pushing the new bit into the storage section of the wire. Finally, we have proven the efficacy of this system inside an array of 7 nanowires, opening the possibility to use this configuration in a 3D racetrack memory device composed of an array of thousands of nanowires produced by low-cost and high-yield template-electrodeposition methods.

## 1. Introduction

The huge amount of new data generated every day has caused the need to search for new systems to store all this information in the smallest possible areas. S. Parkin proposed a new storage device called racetrack memory, in which magnetic domains are used to store the information in ferromagnetic stripes along its length [1]. The magnetic domains would act like bits, separated by domain walls (DWs). In this novel system, the DWs are pinned at artificial defects created along the stripe, and the depinning is achieved by the application of spin-polarized current pulses [2]. These pulses initiate the DWs movement, transporting the bits along the stripe reaching the read/write heads, increasing the speed of reading/writing data. In particular, 3D racetrack memories, made of arrays of vertically aligned nanowires, allow us to greatly increase the information density stored on a device.

The artificial defects used to pin the DWs are usually physical constraints placed at regular intervals throughout the magnetic stripe [3,4,5,6], and the distance between them allows us to control the bit size. However, despite the significant advantages of racetrack memories, like the increment in the information density or the high reading speed, this potential new storage device still has some limitations that prevent its implementation. Two of the main challenges that are under study are the need to avoid the Walker breakdown to increase the DW velocity [7,8], and the unwanted local Joule heating created at the notches when applying a current pulse through the wire to move the information pattern [9,10].

The first problem can be solved using cylindrical nanofibers instead of rectangular stripes, where it has been proven that the Walker breakdown limitation disappears [7]. In addition to that, the use of nanofibers presents important advantages in comparison with the stripes: they can be fabricated in arrays by low-cost and high-yield electroplating methods (increasing the storage density), their geometry is easily tuned during the growing process and it is possible to grow segments of different materials along the wire [11,12,13,14].

On the other hand, by using nanofibers instead of stripes it is also possible to solve the second problem mentioned before: the possibility of manufacturing segments with different materials allows the creation of chemical rather than physical constraints [15,16,17,18,19]. Chemical constraints consist of sections within the nanowire where the chemical composition changes with respect to the rest of the sample. If one segment of non-magnetic material is grown in the middle of two magnetic segments, the DW can be pinned at this constraint [20,21]. Its use avoids the presence of physical constraints, so the geometry does not change along the wire and the undesired overheating is eliminated. Many reports can be found on the fabrication of ordered arrays of vertically aligned cylindrical magnetic nanowires with chemical constraints along the wire’s length, such as Ni/Au [22], Ni/Cu [14], Ni_20_Fe_80_/Ni_70_Fe_30_ [16], Co/Au [17], Co/Cu [11], Fe/Cu [23], FeCoCu/Cu [24] and FeGa/Cu [25]. The morphology, chemical composition homogeneity, crystallographic structure and magnetic properties have also been thoroughly studied by scanning and transmission electron microscopy, electron energy loss spectroscopy, electron holography [11], X-ray magnetic circular dichroism photoemission electron microscopy [16,19], magnetic force microscopy [15], and bright-field transmission electron microscopy and Lorentz microscopy [18]. Recent works have also proven the viability of such cylindrical magnetic nanowires with chemical constrains as key elements for magnetic recording, where pinning of the magnetic domain walls was confirmed at the constrains, and depinning accomplished by applying external magnetic fields [16,19].

Considering the easy tuning of racetrack nanowires during the electrodeposition process together with the use of chemical constraints, a novel 3D memory configuration is proposed in this work. The possibility to modify each segment in an individual way allows the creation of functional segments. Therefore, in this work we propose the integration of a writing section along the nanowire to replace the external writing heads. The creation of two different functional sections (writing and storage) along the same track provides a simpler way to write and store information in a high-density storage memory device. The main idea is to create a writing section made of two magnetic segments with different coercive fields, isolated from the storage section by a non-magnetic chemical constraint. By using a writing section that has one segment with a lower coercive field than the rest of the racetrack device allows us to switch its magnetization in an independent and easy way by applying a small external magnetic field. The information written in this first section can then be recorded into the racetrack memory wire by applying a spin-polarized current through the device, allowing us to push (and write) a new bit into the storage section of the track.

This work presents an in-depth study of the viability of this system as a 3D racetrack memory device using micromagnetic simulations. First, we demonstrate the viability of the writing section by simulating three different approaches to modify the coercivity of one segment: (i) physical (tuning the diameter), (ii) chemical (changing the chemical composition) and (iii) structural (adjusting the magnetocrystalline anisotropy). Once the bit has been successfully written using a magnetic field, we demonstrate the recording of the bit into the storage section by applying a spin-polarized current pulse through the nanowire. The use of a spin-current allowed the movement of all the DWs pinned at the chemical constraints along the track towards the adjacent pinning site, proofing the successful recording of the newly written bit without affecting the previously stored information. Finally, we analyze arrays of 3 and 7 nanowires to investigate the effect of neighboring magnetic wires in the bit movement of the central track. Our results show successful writing and storage processes even inside an array of nanowires, opening the possibility to use this system in a 3D racetrack memory device fabricated by industrially scalable methods.

## 2. Numerical Methods

Magnetic simulations were performed using the Object Oriented Micro-Magnetic Framework (OOMMF) project to solve the Landau-Lifshitz-Gilbert (LLG) equation [26]. The LLG equation in the case where the current is aligned with the wire axis is given by [27]:

(1) $$\frac{d\vec{m}}{dt}=\left|\gamma \right|\vec{H_{eff}}\times \vec{m}+\alpha \vec{m}\times \frac{d\vec{m}}{dt}-\left(\vec{u}\cdot \vec{\nabla }\right)\vec{m}$$

In this equation, *γ*, α, $\vec{H_{eff}}$, *t,* $\vec{\nabla }$ and $\vec{m}$ correspond to the gyromagnetic ratio, Gilbert damping parameter, effective magnetic field, time, operator nabla and unit vector of the magnetization, respectively. The velocity *u* is defined as:

(2) $$u=\frac{JPg\mu_{B}}{2eM_{s}}$$

where *J*, *P*, *g*, $\mu_{B}$, *e* and $M_{s}$, are the current density, the spin polarization rate, the g-factor of the electron, the Bohr magneton, the electron charge and the saturation magnetization, respectively. In this work, the current was simulated as pulses, and the external magnetic field was simulated as DC. Both of them were applied along the *x*-axis, which corresponds to the long axis of the nanowires.

Individual and arrays of multi-segmented cylindrical nanowires composed of several magnetic segments (Ni or Co) separated by non-magnetic thin layers, were simulated. The Ni segments consist of pure Ni with an exchange constant of 9 × 10^–12^ J/m, a saturation magnetization of 490 × 10^3^ A/m and no magnetocrystalline anisotropy. On the other hand, the Co segments have an exchange constant of 30 × 10^−12^ J/m and a saturation magnetization of 1400 × 10^3^ A/m. Their magnetocrystalline anisotropy constant varies from zero, for the “Co without anisotropy” segments, to 5.7 × 10^3^ J/m^3^, if the segment is considered as “Co with anisotropy” (corresponding to Co nanowires with an hexagonal close-packed (hcp) crystallographic structure where the magnetocrystalline anisotropy axis is lying along the nanowire’s length). The non-magnetic segments were simulated without saturation magnetization nor exchange constant and will be referred to as Au segments.

The diameter of the nanowires was set to 40 nm, while the length of individual segments changed from 200 nm (for the Ni and Co segments) to 20 nm (for the non-magnetic chemical constraints). A cubic mesh with a unit cell size lower than the Ni and Co exchange lengths (*l*_ex_ = 7.72 nm and *l*_ex_ = 4.94 nm, respectively [26]) is necessary, so a unit cell size of 4 nm was used in all simulations.

## 3. Results and Discussion

The two main challenges of a racetrack memory are the writing process and the bit movement. In this work, we simulate a novel model of a 3D racetrack memory where the use of functional segments eases to write and store information in a single cylindrical nanowire. This research has been performed through the analysis of three different stages: (1) the writing step, (2) the storage process (bit movement), and (3) the effect of neighboring nanowires during the DW movement in a single racetrack. The writing process has been performed by the application of an external magnetic field, and the bit movement has been achieved through a spin-polarized current pulse. Each process will be explained in detail in the following sections.

### 3.1. Writing Process

The writing process can be defined as the stage where one new bit is recorded. From a magnetic point of view, it means that the system should be able to inverse the magnetization of one segment of the nanowire in this stage, maintaining invariable the magnetization of the other segments.

In the proposed model, the memory recording is performed by the application of a magnetic field. The change in magnetization in only one segment of the entire nanowire is solely achieved when it presents any difference from the rest of the segments that reduces its coercive field. This difference can be physical, chemical, or structural:

- Physical: By modifying the nanowire’s diameter one can easily tune its coercivity [28,29]. A segment with a larger diameter than the rest of the nanowire reverses its magnetization at lower applied magnetic fields (smaller coercivity). This modification can be easily scaled-up by adjusting the anodization conditions during template fabrication [13]. For example, Salem et al. reported the fabrication of cylindrical Ni_80_Fe_20_ nanowires with modulated diameters by enlarging the pore diameter in a chemical bath prior to the final anodization process [30]. On the other hand, Méndez et al. combined electrochemical anodization, atomic layer deposition and pore widening steps to fabricate diameter-modulated FeNi nanowires with sharp transition regions between the two segments of different diameters [28].
- Chemical: The deposition of two elements with different coercive fields (e.g., Ni and Co [31,32]) eases the creation of a writing segment without the need of modifying the initial template. Their fabrication follows a simple multi-step electroplating process with varying electrolyte compositions, as previously described elsewhere [15,18,33].
- Structural: By tuning the deposition parameters one can modify the crystallographic structure of the deposits, which, for elements with high magnetocrystalline anisotropy along the wire axis, will vary the coercive field. For example, the modification of the electrolyte pH during Co deposition can modify its structure from a face centered-cubic (fcc) one (with low magnetocrystalline anisotropy) into a hcp one (with high magnetocrystalline anisotropy) [34,35]. Other reports have also proved the fine-tuning of the crystallographic structures of Co nanowires by a pulsed-controlled electrochemical growth [36,37,38]. In particular, Montazer et al. reported enhanced coercivities for large-diameter Co nanowires by adjusting the crystallographic structure of the deposits [36].

**Figure 1:**
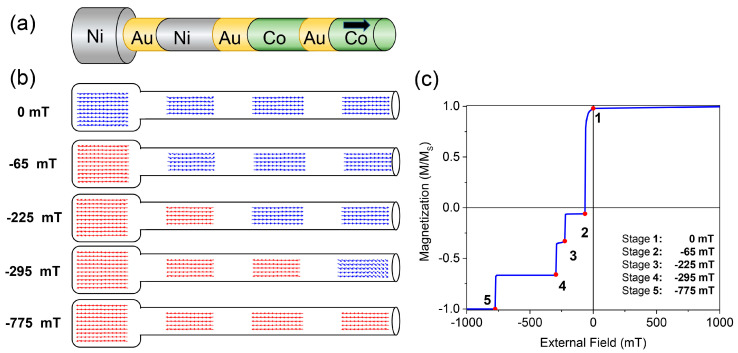
(**a**) Schematic representation of the nanowire with the four different segments. From left to right: Ni with larger diameter, Ni with smaller diameter, Co without magetocrystalline anisotropy and Co with magetocrystalline anisotropy. (**b**) Different stages of the magnetic simulation during the demagnetization process. (**c**) Hysteresis loop of the magnetic simulation. Red dots indicate the stages showed in (**b**).

The applied magnetic field used to switch the distinctive segment should be high enough to reverse its magnetization but small enough to avoid the inversion of magnetization in the other segments. To study all the potential differences mentioned before, the switching field of each possible segment has been estimated by simulating a single nanowire with four isolated magnetic segments: one of Ni with a larger diameter (80 nm instead of 40 nm), two with the same diameter but different elements (Ni and Co with negligible magnetocrystalline anisotropies and 40 nm in diameter); and a Co segment with an hcp crystallographic structure that induced a magnetocrystalline anisotropy along the wire’s length. To avoid the propagation of the magnetic domains from one segment to the adjacent one, non-magnetic Au segments of 150 nm were intercalated between the magnetic ones. Figure 1a shows a representation of the simulated nanowire.

The demagnetization process of the different segments is schematically represented in Figure 1b, where each stage corresponds to one drop in the hysteresis loop of the nanowire (Figure 1c). As can be observed in Figure 1b,c, all the segments maintain their magnetization in the remanence stage (0 mT), indicating that they could be used to store information, which is essential in a racetrack memory device. When a negative magnetic field is applied, the first segment that inverses its magnetization, i.e., the segment with the lowest coercive field (*H*_c_), is the one with a larger diameter (*H*_c_ = 65 mT). As the external field increases (towards negative values), the next segment inversing its magnetization is the Ni-segment with a diameter of 40 nm. The difference in the exchange stiffness constant between Ni (9 × 10^−12^ J/m) and Co (30 × 10^−12^ J/m) causes the demagnetization of the Ni-segment at a lower field than in the case of the Co-segment. However, since both segments present the same diameter and zero magnetocrystalline anisotropy constant, the difference between their coercive fields is less than 100 mT (225 vs. 295 mT). The last segment that inverses its magnetization is the Co-segment with a large magnetocrystalline anisotropy constant (along the wire’s length), exhibiting a coercive field of 775 mT (Figure 1c).

**Figure 2:**
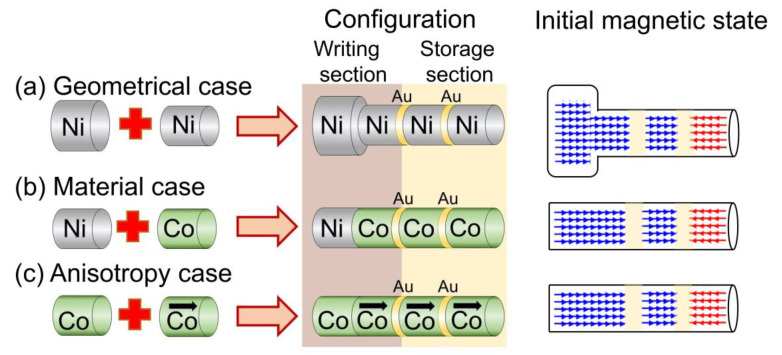
Scheme of the three different nanowires simulated during this work. Each nanowire is composed of a writing section (combining a soft and a hard magnetic segment) and a storage section (made of two hard magnetic segments). The hard magnetic segments are separated by non-magnetic Au layers with 20 nm in thickness (chemical constraints).

As explained before, the combination of segments with different coercivities would ease the magnetic modification of selective sections, maintaining invariant the other ones. The main idea is thus to create a writing section along the wire composed of two magnetic segments with different coercivities: a soft (low coercivity) magnetic segment that is different from the rest of the wire, and a harder (higher coercivity) magnetic segment, equal to the rest of the bit-storage segments. By being adjacent to each other, the reversal of the soft segment would induce the switching of the harder segment at a lower applied field than its coercivity. This first part of the wire (writing section), would then be separated from the rest of the wire (storage section) by a thin non-magnetic layer (chemical constraint), so it should show a lower coercive field than the rest of the nanowire. Based on the results shown in Figure 1, three different possibilities for a racetrack memory are proposed (Figure 2):

- Geometrical case: The diameter of the soft magnetic segment is bigger. In this case, all the magnetic segments are made of the same element, Ni.
- Material case: The first segment is made of Ni (*H*_c_ = 225 mT), while the rest of the magnetic segments are made of Co (*H*_c_ = 295 mT).
- Anisotropy case: All the magnetic segments are made of Co, but the first magnetic segment is the only one with no magnetocrystalline anisotropy.

**Figure 3:**
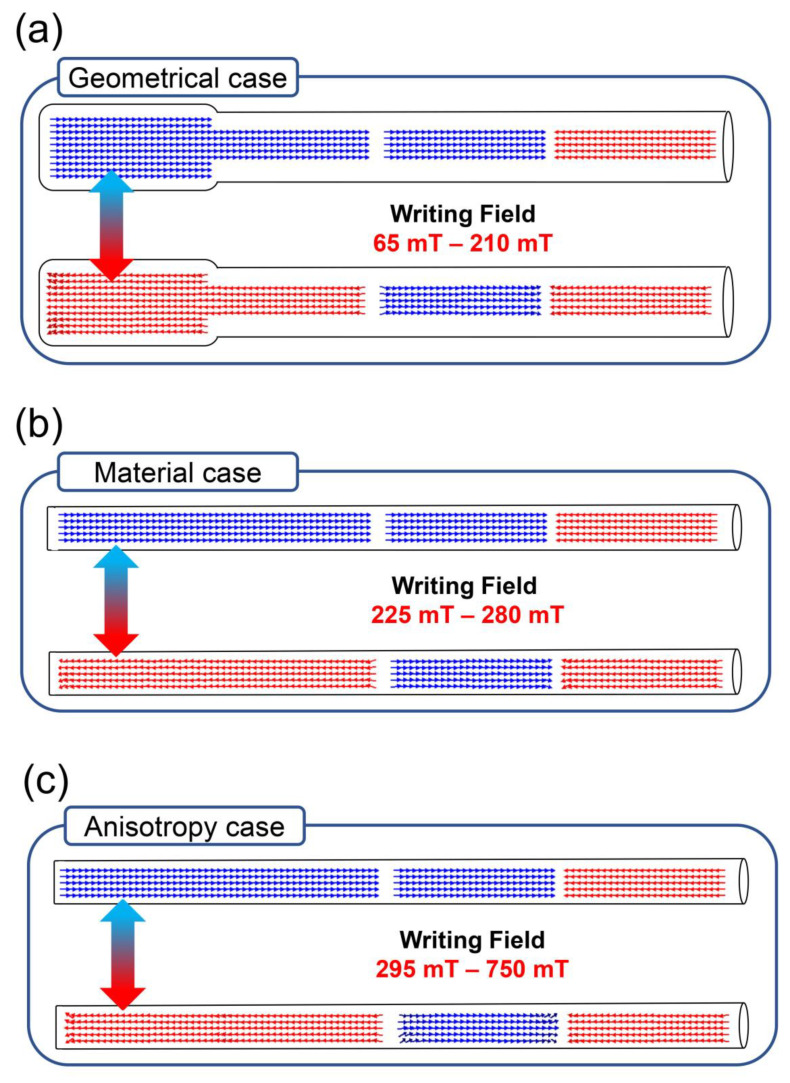
Representation of the low energy state of the (**a**) geometrical, (**b**) material and (**c**) anisotropy cases after applying the magnetic field during the writing process. The lowest and the highest magnetic fields that can be applied to switch from the top stage to the bottom of each case is called writing field.

The lowest magnetic field that can be applied to switch the magnetization of the writing section in each case will be determined by the coercive field of the soft-segment. At the same time, the applied magnetic field cannot be higher than the coercive field of the hard-segments, since it would clean all the bits recorded in the nanowire. Figure 3 shows the range of “writing fields” that can be used in each case.

The three nanowires simulated can successfully reverse the magnetization of the writing section when applying a magnetic field in the range of 65–210 mT (geometrical case), 225–280 mT (material case) and 295–750 mT (anisotropy case) and maintaining invariant the magnetization of the rest of the nanowire. Independently of the success of the writing process, it can be observed that the highest magnetic field that can be applied to switch the magnetization in the writing section is slightly lower than the coercive field of the storage segments previously simulated (Figure 1b,c). Since the non-magnetic Au segments in these nanowires are thinner than in the previous case (20 nm instead of 150 nm), the magnetic domains can propagate to adjacent segments if the applied field is too high. However, if the applied magnetic field is in the range shown in Figure 3, the magnetic configuration of each segment cannot cause demagnetization of the adjacent one, so the information of the storage section stays invariant during the writing process. By maintaining invariant the length of the Au segments along the nanowire, the result presented here could be extrapolated to a nanowire with as many segments as desired, independently of the magnetic configuration of the system.

This result proves the effectiveness of the proposed model, where the construction of a single nanowire with two different types of segments together with the use of chemical constraints eases to write a bit in one segment without any variation in the rest of the track, by an external magnetic field. Once the bit has been written, the next step is its movement along the nanowire to record the information into the storage section of the racetrack. Based on previous experimental results, where a spin current was proven as a successful method to displace DWs in a stripe [1,2,4,39,40,41,42,43], the movement of the bits has been performed by the application of a current pulse.

### 3.2. Bit Movement

The bit movement is produced by applying a spin-polarized current pulse along the nanowire, which causes the propagation of the magnetic domains from a segment to the adjacent one. All the bit displacements should occur at the same time. A delay in the onset of one bit displacement could produce an overwriting in a storage segment before it is able to transmit its information to the following one. In order to avoid this delay, the transmissions must be produced between equal segments. For that reason, the scheme in Figure 2 shows that the writing sections of the three cases are composed of two segments: one soft-segment with a lower coercive field (necessary to switch the magnetization) and, attached to it, a hard-segment with the same characteristics as the storage-segments. The latter is required for the magnetization propagation from the first segment to the following one to occur at the same time than the rest of the nanowire.

**Figure 4:**
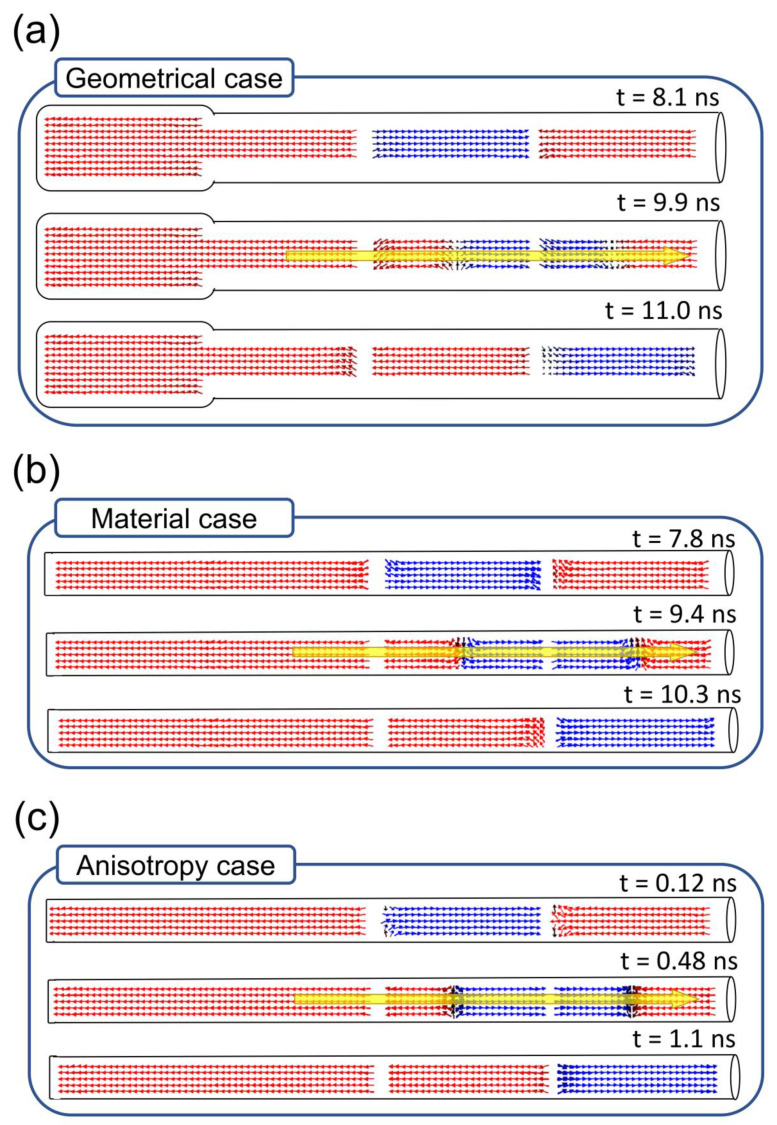
Representation of the (**a**) geometrical, (**b**) material and (**c**) anisotropy cases during the bit movement. The time necessary to depin the domain wall (top) and to complete the bit movement (bottom) is shown in each case. The time indicated in each case corresponds to the time elapsed since the current pulse started.

The propagation of the magnetic domains caused by the application of a spin-polarized current is only possible if the used current density is high enough to depin the DWs from the chemical constraints. This is directly related to the velocity u presented in the LLG equation (Equation 1). Therefore, the magnetic domains will only propagate to the adjacent segment if the vector u is high enough. Previously reported *u* values are in the range of 100–600 m/s [27,44]. In this work, two different velocities have been used to displace the magnetic domains, attending whether the storage segments have magnetocrystalline anisotropy or not. A velocity of 100 m/s was used in the geometrical and material cases and 300 m/s in the anisotropy case, due to the extra difficulty observed in the propagation of magnetic domains in materials with magnetocrystalline anisotropy. However, although the velocity u in the first two cases is the same, it is important to remark that the vector u is directly related to the current density and inversely dependent on the saturation magnetization of the material (Equation 2). Therefore, the current density necessary to obtain the velocity mentioned before in an experimental work would be different in each case. The values used in this manuscript would correspond to an equivalent current density in the order of *J* ≈ 10^11^ A/m^2^. Figure 4 shows the magnetic domains propagation using the velocities mentioned before.

The results presented in Figure 4 prove the success of the model proposed, achieving the propagation of the magnetic domains independently of the case exposed. The different velocity used in the geometrical, material and anisotropy cases explains the great disparity observed in the propagation time: while the anisotropy case only needs 1 ns to finish the propagation of the magnetic domain from one segment to the adjacent one, 10.3 and 11 ns are needed to observe the same propagation in the material and the geometrical cases, respectively. Despite the time needed or the velocity used, the DW formed inside each segment during the domain movement can be described in the three cases as a transversal like DW. This result is in good agreement with Castilla et al. [20], where it is reported that the pinning of a DW in a chemical constraint (the Au segment in this case) transforms any kind of DW into a transversal DW, which is also observed in these simulations. The movement of the transversal DW under the application of a spin current has been studied by the animation of the storage process in the geometrical case, which is shown in Supplementary information Video S1. The 3D animation shows a first stage where the transversal DW is formed and a second stage where it is propagated along the segment. These two stages can be well-differentiated by observing the rotation of the DW. During its formation, the DW shows a rotation around the segment, which disappears once the DW is stable and starts its linear propagation throughout the segment.

Direct comparison between Figure 3 and Figure 4 proves how the use of a 20 nm-thick Au layer to isolate the magnetic segments successfully manages to pin the domain wall at the chemical constraint and, at the same time, allows its depinning when a spin-current is applied through the nanowire.

The simulated starting magnetic configuration in this section was chosen with the purpose of being able to observe the DW propagation along the two storage sections at the same time. Nevertheless, since the initial magnetic configuration does not influence the onset of the DW movement, all the possible magnetic configurations would initiate the depinning process at the same time. In an up-scale process with tens of storage sections in a single nanowire, the expected behavior in this longer nanowire would be the same than that observed in Figure 4.

Once the proposed model has been proved as an interesting option for a racetrack memory, the next step is to study its viability as a 3D storage device. For that, we will investigate the bit movement in a single nanowire inside an array of several nanowires, as magnetostatic interactions between neighboring elements are known to influence the magnetization reversal process [45,46,47].

### 3.3. Arrays of Nanowires as 3D Racetrack Memories

Two different arrays with 3 and 7 nanowires (hexagonal array), respectively, have been simulated to investigate the effect of neighboring magnetic wires in the bit movement of the central wire. For simplicity, and given the similarity of results, in this section only the geometrical case has been considered.

**Figure 5:**
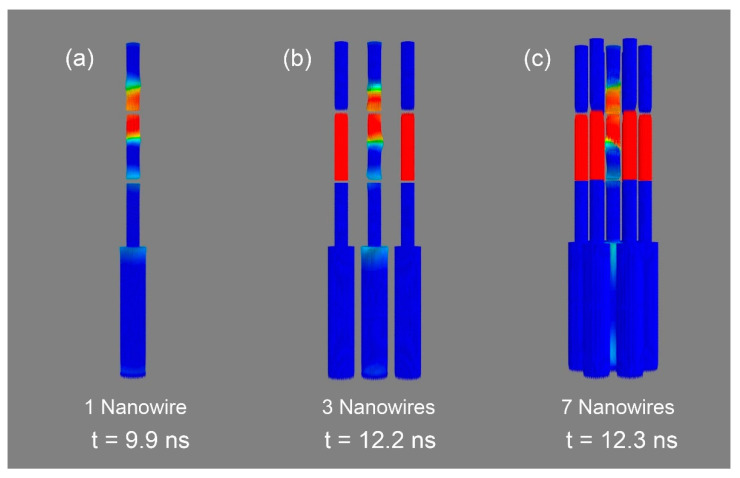
3D representations of the bit movement in (**a**) a single nanowire and in the central wire of an array with (**b**) 3 and (**c**) 7 nanowires. The time indicated in each case corresponds to the time elapsed since the current pulse started.

As all the nanowires are identical, the writing-section of all of them will switch their magnetization under similar applied fields [45]. The presence of neighboring wires will reduce the overall switching field but will still maintain a similar writing field range as displayed in Figure 3. On the other hand, the bit movement of each nanowire should be performed individually, so it is necessary to investigate the possible influence of the neighboring nanowires when a spin current is applied to the central one. For that, the spins of the external nanowires were fixed during the simulation of the bit-movement in the central nanowire. 3D representations of the three different configurations compared in this section are plotted in Figure 5. With the aim of comparing the bit movement under the same conditions, the velocity u in all cases has been set to 100 m/s. The center-to-center distance between the nanowires was set to 104 nm, based on previous experiments using nanoporous alumina templates [47,48].

**Figure 6:**
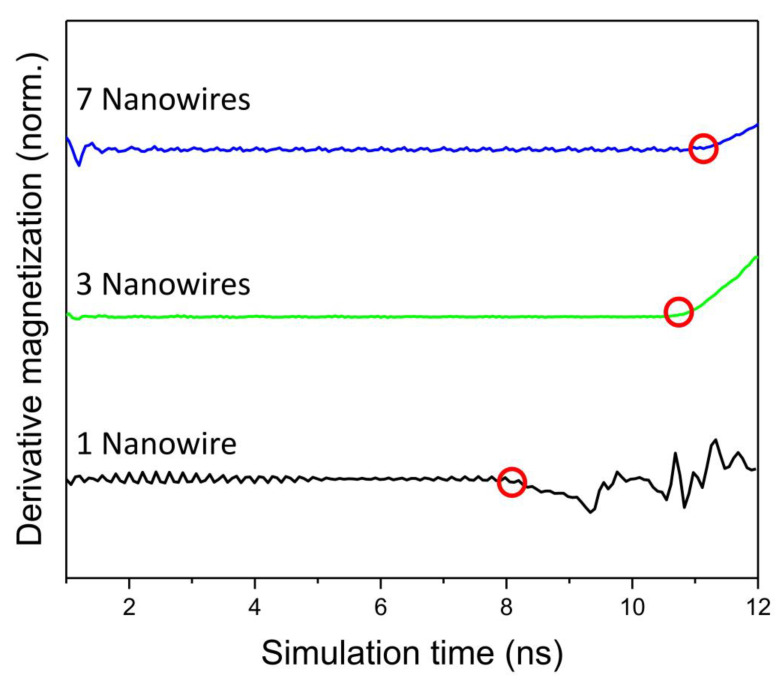
Derivative of the magnetization along the wire’s length (*M_x_*) during the current pulse in the case of 1, 3, and 7 nanowires. Red circles show the moment of the depinning of the domain wall.

Simulations show that magnetic domains can propagate from one segment to the adjacent one even when in an array with 3 or 7 nanowires, but a longer pulse time is needed. The presence of neighboring nanowires thus delays the bit movement. Figure 6 shows the derivate of the magnetization value along the wire (*M_x_*) for each case. It can be clearly seen how the onset of the bit movement increases from 8.1 to 10.8 ns in the case of 1 and 3 nanowires, respectively, and reaches 11.2 ns in an array of 7 nanowires. These results show a significant delay of almost 3 ns in the depinning of the DW when the central nanowire has others surrounding it, which is a critical difference that should be considered in the experimental development of a future 3D racetrack memory device. However, it can be observed that the number of nanowires around it only slightly modifies this onset (10.8 vs. 11.2 ns), so the density of nanowires in the array does not seem to be a key factor in the development of the 3D racetrack memory. As in the previous case where it was analyzed a single nanowire, the magnetic configuration of the surrounding nanowires does not introduce any modification in the depinning process.

**Figure 7:**
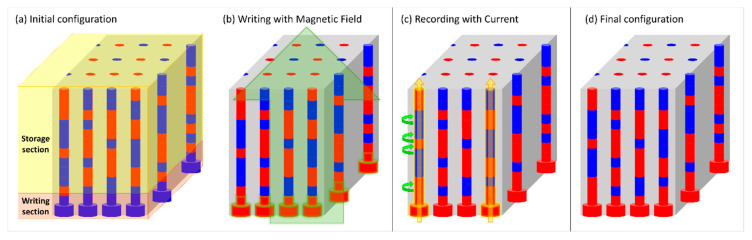
Schematic representations of the (**b**) writing and (**c**) recording processes in a 3D racetrack memory device changing the stored information from (**a**) initial to (**d**) final configuration. Red (blue) segments correspond to magnetic domains pointing up (down). Yellow arrows in (**c**) indicate the application of a spin-polarized current along the respective wires.

To conclude, we present in Figure 7 a schematic representation of a 3D racetrack memory device based on the novel design proposed in this work. The device is composed of an array of vertically aligned cylindrical nanowires (racetracks). Each track is made of a storage section (capable of recording 11 bits of information) and a writing section (comprising an additional information bit and a lager diameter segment (soft-segment)). By applying a small external magnetic field, the magnetization of the writing section reverses while keeping the initial configuration of the storage section (no data is lost between Figure 7a,b). To record the new information into the memory device, a spin-polarized current is applied to selected tracks (yellow arrows in Figure 7c). From the experimental point-of-view, this could be performed following a nano-identation procedure described elsewhere [49,50] optimized through different automatic compilation frameworks [51] or through reinforced learning [52]. Although existing characterization techniques do not yet allow a fast access of individual tracks in a vertical array, this type of equipment is constantly being updated with innovative designs and functionalities, so these measurements may become feasible in the near future. Meanwhile, combinations of nano-lithographed patterns with atomic force microscopy nano-identation techniques would allow preliminary studies of the feasibility of such 3D nano-memory devices. At the end of the process, we were able to simultaneously record two bits of information into two different tracks (Figure 7d). Supplementary information Figure S1 shows an animated representation of the writing and storage processes in which several different bits are recorded at the same time.

## 4. Conclusions

In this work, a novel 3D racetrack memory configuration based on functional segments inside cylindrical nanowire arrays has been proposed. The integration of the writing and the storage sections inside the same track has been studied through micromagnetic simulations, proving the success of this novel configuration without the use of external writing heads.

The incorporation of the writing and the storage sections together has been made possible by growing two kinds of segments with different magnetic properties in a single nanowire. Micromagnetic simulations of cylindrical nanowires based on magnetic segments of Ni and Co have demonstrated the possibility to reduce the coercive field of one segment of the nanowire by different modifications: physical, chemical, or structural.

The ability to grown two kinds of magnetic segments with different coercivities has been used to build a writing section in the nanowire. This section has been made by the combination of a soft (low coercivity) magnetic segment with a harder (higher coercivity) magnetic segment, equal to the rest of the bit-storage segments (storage section). By being adjacent to each other, magnetic simulations have shown how the reversal of the soft segment can induce the switching of the harder segment at a lower applied field than its coercivity, writing the new bit. The use of a thin non-magnetic layer (chemical constraint) between the writing section and the rest of the wire (storage section) avoids the propagation of the magnetization reversal to the following segments; keeping the storage section invariant during the writing process and preserving the information stored in the rest of the nanowire.

While the bit recording process was performed under the application of an external magnetic field, the store of the new bit was achieved using spin-current pulses. Magnetic simulations have shown how the application of spin-current pulses causes the domain wall movement from one magnetic segment to the adjacent one, pushing the new bit into the following section of the wire and allowing its storage. The simulations have proven that the presence of chemical constraints along the nanowire stops the inversion of the magnetization during the writing process and, at the same time, allows the domain wall movement when a spin-polarized current pulse is applied.

Finally, we studied the effect of neighboring nanowires when a spin current is applied to the central nanowire, proving that it is also possible to write and store a bit in a nanowire inside an array. The neighboring nanowires cause a delay in the domain wall movement when a spin-current is applied, but their presence does not prevent the storage process.

These results suppose a breakthrough in the research of 3D racetrack memory configuration. While most of the studies are based on the research of rectangular stripes where it is not possible to grown functional sections, this work presents a novel configuration where it is possible to write and record bits in cylindrical nanowires. The use of nanowires instead of stripes enables the growth of thousands of wires in a single deposition, reducing the cost and increasing the information density of the memory.

## Supplementary Materials

The following are available online at https://www.mdpi.com/2079-4991/10/12/2403/s1, Video S1: Storage-Process, Animated Figure S1: 3D-NanoMemory.
