# Supplementary figures and images for: A Novel Design of a 3D Racetrack Memory Based on Functional Segments in Cylindrical Nanowire Arrays
Source: Nanomaterials (Basel). 2020 Dec 1;10(12):2403. doi: 10.3390/nano10122403 (PMC7761019; doi:10.3390/nano10122403)

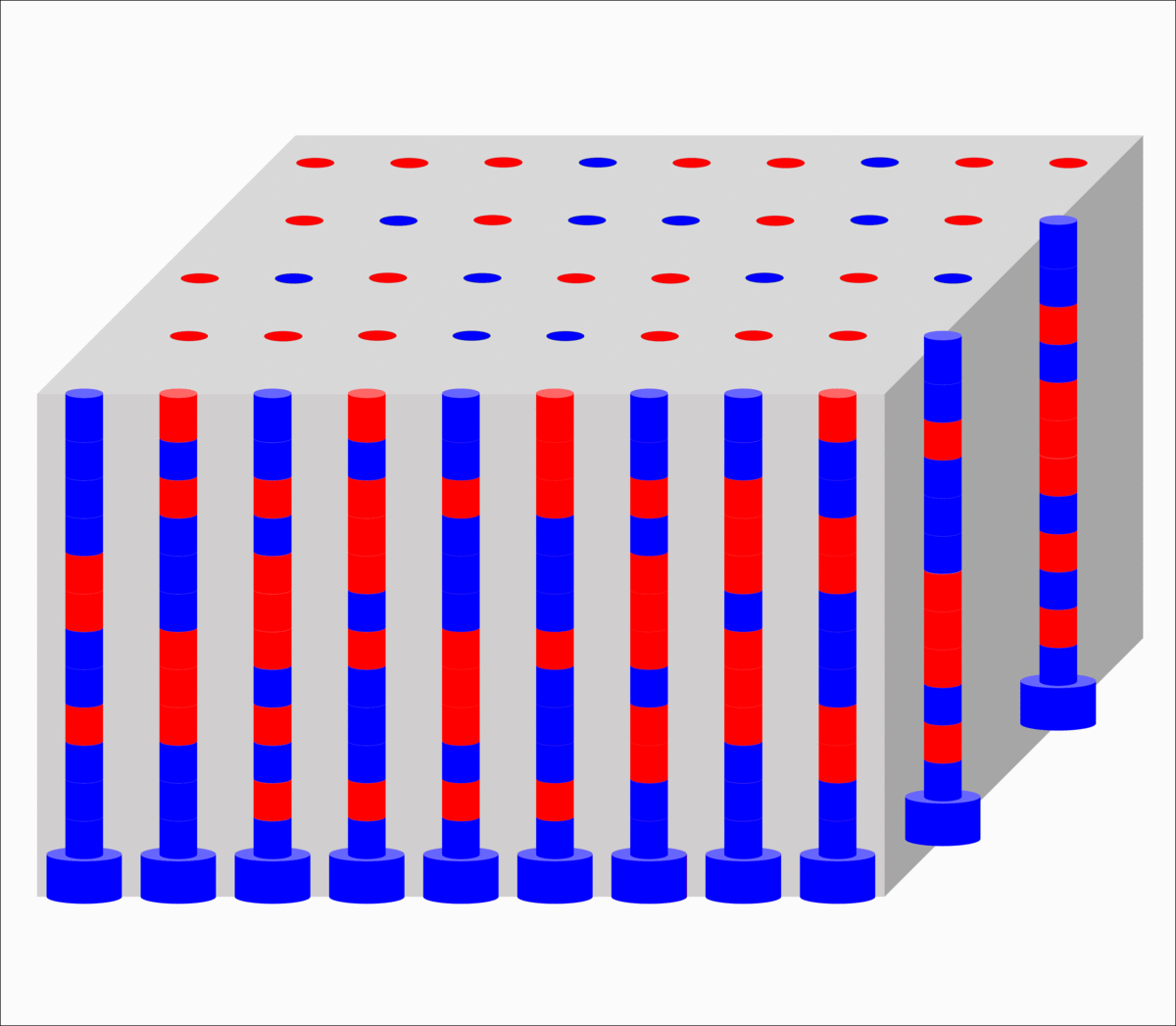

Supplement: Supplementary file 1 [file nanomaterials-10-02403-s001.zip › Figure S1. 3D-NanoMemory.gif]
